# Protocol for the Development of The Australian Motor Neurone Disease (MND) Guideline

**DOI:** 10.1002/gin2.70055

**Published:** 2025-12-10

**Authors:** Cindy Stern, Danielle Pollock, Timothy Barker, Grace McKenzie Holland, Sabira Hasanoff, Nipun Shrestha, Ines Semendric, Lemma Negesa Bulto, Sitasma Sharma, Anna Fragkoudi, Sebastian Cole Facchin‐Young, Abigail Molly Day‐Sharman, Lara Stollery, Jay Beasley‐Hall, Camille Schubert, Lynne Giles, Tracy Merlin, Steve Vucic, Jo Whitehouse, Zachary Munn

**Affiliations:** ^1^ Health Evidence Synthesis, Recommendations and Impact (HESRI), School of Public Health, Faculty of Health and Medical Sciences The University of Adelaide Adelaide Australia; ^2^ School of Biomedicine, Faculty of Health and Medical Sciences The University of Adelaide Adelaide Australia; ^3^ University Library The University of Adelaide Adelaide Australia; ^4^ Adelaide Health Technology Assessment (AHTA), School of Public Health, Faculty of Health and Medical Sciences The University of Adelaide Adelaide Australia; ^5^ School of Public Health, Faculty of Health and Medical Sciences The University of Adelaide Adelaide Australia; ^6^ Brain and Nerve Research Centre, Concord Clinical School The University of Sydney Sydney Australia; ^7^ MND Victoria Canterbury Australia

**Keywords:** amyotrophic lateral sclerosis, GRADE, guideline, lived experience, care management, implementation, Lou Gehrig's disease, motor neurone disease, neurodegenerative diseases

## Abstract

**Introduction:**

Motor neurone disease (MND) is a rare but devastating neurodegenerative condition that results in progressive muscle weakness, disability, and eventual respiratory failure. In Australia, the impact of MND is significant, and there is a pressing need for a high‐quality, locally relevant guideline to support equitable, person‐centred care.

**Questions:**

This guideline aims to address key clinical and care‐related questions that arise across the MND trajectory. It will identify the most pressing topics and uncertainties in MND management through interest holder consultation and structured prioritisation.

**Methods:**

The guideline will be developed using the GRADE (Grading of Recommendations, Assessment, Development and Evaluation) approach and will align with standards set by the National Health and Medical Research Council (NHMRC) and the Guidelines International Network (GIN). Governance will include a multi‐tiered structure with lived experience representation. Implementation planning and evaluation will be integrated throughout, with a planned transition to a living guideline model supported by an ongoing evidence surveillance strategy.

## Summary

- Motor neurone disease (MND) is a rare neurodegenerative disease that currently has no cure. Management focuses on symptom management to improve life quality.
- There is currently no Australian guideline for MND.
- The guideline will follow GRADE methodology.
- A six‐stage process for prioritising scope, topic and questions will be utilised.
- A multi‐tiered governance structure will be established to ensure guideline co‐creation.
- Implementation and evaluation activities will be considered simultaneously with guideline development.
- The guideline will be designed to facilitate transition to a living model.

## 1 Introduction

Motor neurone disease (MND) is a progressive neurodegenerative disorder marked by the deterioration of motor neurons in the brain and spinal cord. It leads to muscle weakness, disability, and eventual respiratory failure [1, 2, 3]. In Australia, approximately 1500–2000 individuals live with MND at any given time, with two people dying from the disease each day [3, 4, 5]. Despite its relatively low prevalence, MND carries a significant individual, social, and economic burden due to its rapid progression and lack of a cure [6].

There is a critical need for a high‐quality guideline to support optimal management of MND. Well‐implemented guidelines can drive earlier diagnosis, multidisciplinary care, and timely interventions that align with the values and preferences of people living with MND [7, 8, 9, 10]. Importantly, they support equity in care and the integration of emerging evidence into practice [11, 12]. Although guidelines exist that address MND care, they vary in quality and there is currently no guideline available that has been developed for an Australian context [13, 14].

This protocol outlines the proposed methodology and methods for developing an Australian MND guideline. Development of the guideline is being funded by FightMND [15], for a period of 2 years. It draws on internationally recognised methods and prioritises interest holder engagement [16, 17]—particularly those with lived experience.

## 2 Approach

The guideline will follow the GRADE (Grading of Recommendations, Assessment, Development and Evaluation) approach [14], and align with standards from the National Health and Medical Research Council (NHMRC) [18] and the Guidelines International Network (GIN) [19, 20]. The guideline was registered on the GIN Library and the PREPARE (Practice guideline REgistration for transPAREncy) Registry and was approved for consideration for the NHMRC approved guideline programme on 7 November 2024. Any important information pertaining to the guideline will be made available via the OSF project space https://osf.io/c2ezm/.

**Figure 1:**
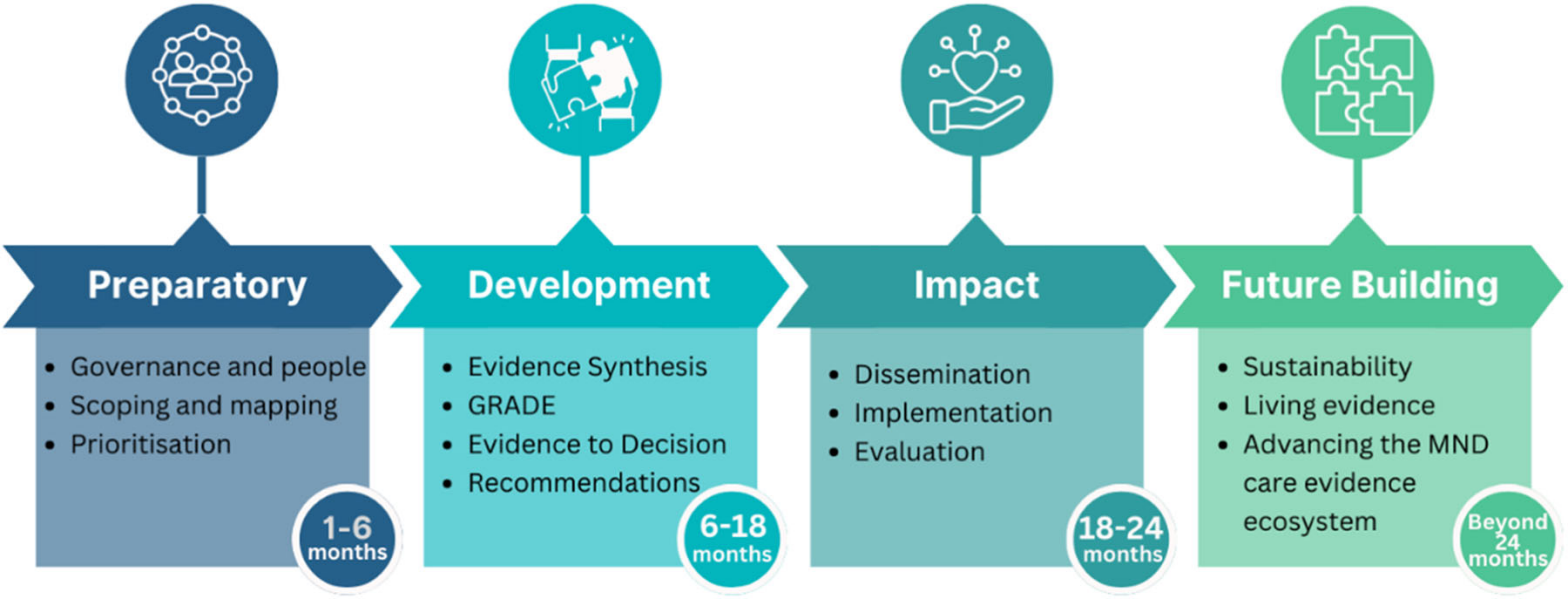
Overview of the overlapping phases for the Australian Motor Neurone Disease (MND) guideline project.

Development of the guideline will follow a four‐phase approach (see Figure 1).

The preparatory phase (Phase 1; 6 months duration) will involve formalising the governance structures and memberships of the guideline groups, mapping previous guidelines and the current MND research landscape, and prioritising guideline questions. Phase 2 will be the development phase (18 months duration) where a series of evidence syntheses projects will be undertaken to answer prioritised topics identified in phase 1 and will involve the creation of GRADE Evidence Profiles [21] and the use of the GRADE Evidence‐to‐Decision frameworks [22] to inform development of recommendations. The implementation and evaluation phase (Phase 3; 6 months duration) involves developing and acting on plans to disseminate, implement and evaluate the guideline ensuring uptake and impact while Phase 4 (phase currently seeking funding) involves transitioning guideline efforts to a living model and advancing the MND care evidence ecosystem.

## 3 Governance

**Figure 2:**
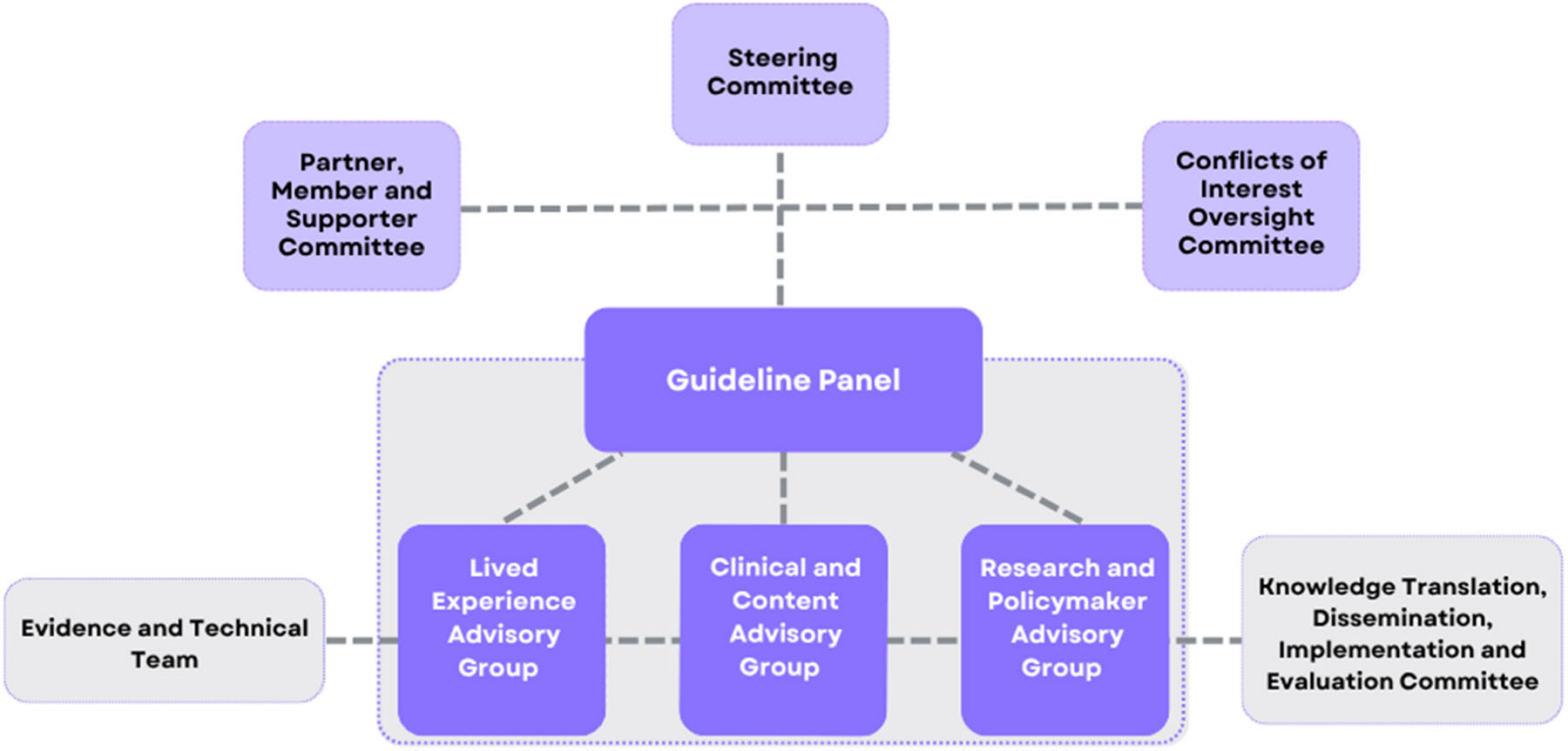
Motor Neurone Disease (MND) guideline governance structure.

A multi‐tiered governance structure has been proposed to promote active inclusion of all interest holders in the guideline development effort and will consist of the following groups (Figure 2):

- Project Steering Committee: Oversees project execution.
- Conflicts of Interest (CoI) Oversight Committee: Provides independent advice on matters relating to CoI of members involved.
- Guideline Development Panel: The main decision‐making body who finalises scope/topics, develops and votes on recommendations, and is responsible for the guideline content.
- Advisory Groups: Three advisory groups (Lived Experience, Clinical and Content, Research and Policymaker) providing feedback and advice throughout all steps involved in the guideline development process.
- Partner, Member and Supporter Committee: Engages key organisations across the Australian MND landscape to ensure they are involved in the effort and to support dissemination efforts.
- Knowledge Translation, Dissemination, Implementation and Evaluation Committee: Focuses on the successful dissemination, implementation and evaluation of the guideline.
- Evidence and Technical Team: Leads all evidence reviews and project coordination.

People with lived experience will be at the forefront of the guideline effort and will be represented across different governance groups. Representation from equity deserving populations such as Aboriginal and Torres Strait Islander peoples and those in regional and remote areas will be actively sought to ensure all perspectives are considered. A Culturally and Linguistically Diverse Communities Advisor and an Aboriginal and Torres Strait Islander Cultural Advisor will be appointed to ensure the guideline is inclusive, culturally sensitive and appropriate for diverse populations.

## 4 Methods

This guideline will adhere to the NHMRC Guidelines for Guidelines [23], which provides overarching principles for rigorous and transparent guideline development. GRADE will be used as a structured approach to assess the quality of the evidence and the strength of the recommendations, ensuring decisions are explicit and reproducible [24]. Where feasible, we will apply the GRADE‐ADOLOPMENT approach, which allows guideline developers to follow the GRADE process of developing guidelines more efficiently by leveraging, adopting, and adapting existing evidence‐based guidelines [25]. The methods will follow the GIN McMaster checklist [20] to guide interest holders engagement, question formulation and evidence‐to‐decision processes. The guideline's methodological quality will be evaluated against the Appraisal of Guidelines for Research and Evaluation II (AGREE II) criteria (Note: AGREE III is under development. Should it be released during the course of guideline development, the team will assess its suitability for use in the project) [26]. Finally, reporting will align with the RIGHT (Reporting Items for practice Guidelines in HealThcare) statement [27], to ensure completeness, clarity and transparency.

### 4.1 Evidence Synthesis Projects

1. Conducting a scoping review of previous guidelines to establish what guidelines currently exist [13] and whether they can be considered for adolopment [25].
2. Develop a living evidence and gap map on all MND care interventions to facilitate a centralised repository of evidence to inform future systematic reviews per prioritised PICO.
3. Conducting systematic reviews of the evidence related to prioritised clinical questions. The living evidence and gap map will be used to identify existing systematic reviews that address the question/s. These reviews will be assessed for credibility [25] and updated if necessary. If no suitable reviews are identified or existing reviews are not considered credible, de novo systematic reviews will be conducted.
4. Conducting a systematic review of values and preferences to inform outcomes to be considered in reviews.
5. Conducting a qualitative review on the experiences of living with MND.
6. Where evidence is limited or does not exist for a particular PICO, we will follow the expert evidence approach and/or seek appropriate registry data to inform recommendations [28] or indirect evidence when judged appropriate by the panel. Members of the Groups, Committees, and Panel will be engaged to provide expert evidence. Where topic‐specific expertise is lacking within the governance structure, external individuals with specialised knowledge will be consulted. Inclusion criteria for invited experts will include relevant clinical or research experience in MND, publication record, and active involvement in MND care or research networks. The number of experts invited will be determined based on the scope of each topic/question to ensure adequate representation. Expert input will be collected primarily via structured online surveys, designed to gather information on outcomes, clinical experience, and implementation considerations.

### 4.2 Scope and Prioritisation

Questions will be informed by interest holder input, lived experience, and identified evidence gaps, and will be framed using the PICO (Population, Intervention, Comparator, Outcome) format. To determine what content will be covered in the guideline a prioritisation process will be undertaken consisting of the following stages:

1. Scoping review of existing guidelines.
2. Broad interest holder consultation (online survey of the Australian MND community).
3. Kick‐off meeting prioritisation workshop.
4. Refinement and finalisation of scope, topics, and questions.
5. Final approval by the guideline panel.
6. Preparation for systematic reviews.

While individual questions are currently being finalised, the panel has voted to focus on the following three scope areas in the first iteration of the guideline: Recognition, Referral and Diagnosis; Clinical Assessment and Care Management; and Models of Care.

### 4.3 Evidence to Decision (EtD)

GRADE provides a stepwise process for framing questions, selecting outcomes and rating their importance, evaluating the evidence, and considering evidence together with the values and preferences of patients and society to arrive at recommendations [21, 24, 29, 30]. After synthesising the evidence in a systematic review for each prioritised question, the results and GRADE certainty rating will be presented in a GRADE Evidence Profile.

Where possible and reasonable, the GRADE‐Adolopment approach will be applied to inform the adoption, adaptation, or de novo development of recommendations for the guideline [25]. The following criteria will be used to determine suitability:

- Published in the past 5 years (2020 onwards) or in the process of being published;
- Addressed clear research questions (contained all Population, Intervention, Comparator and Outcome [PICO] elements);
- Followed the GRADE process;
- Allows for updating (provides access to full systematic reviews and provides full access to the search strategy);
- Included existing and accessible GRADE tables and summaries of findings; and
- Completed a risk‐of bias assessment/formal critical appraisal.

The adaptation process includes taking local contextual factors into consideration and modifying the recommendations accordingly. Where recommendations are identified that align with the specified PICO questions, assessments will be made regarding whether they can be adapted or adopted. Ideally, recommendations identified will have an evidence profile and EtD table included; if not, EtDs may need to be constructed with panel input.

**Figure 3:**
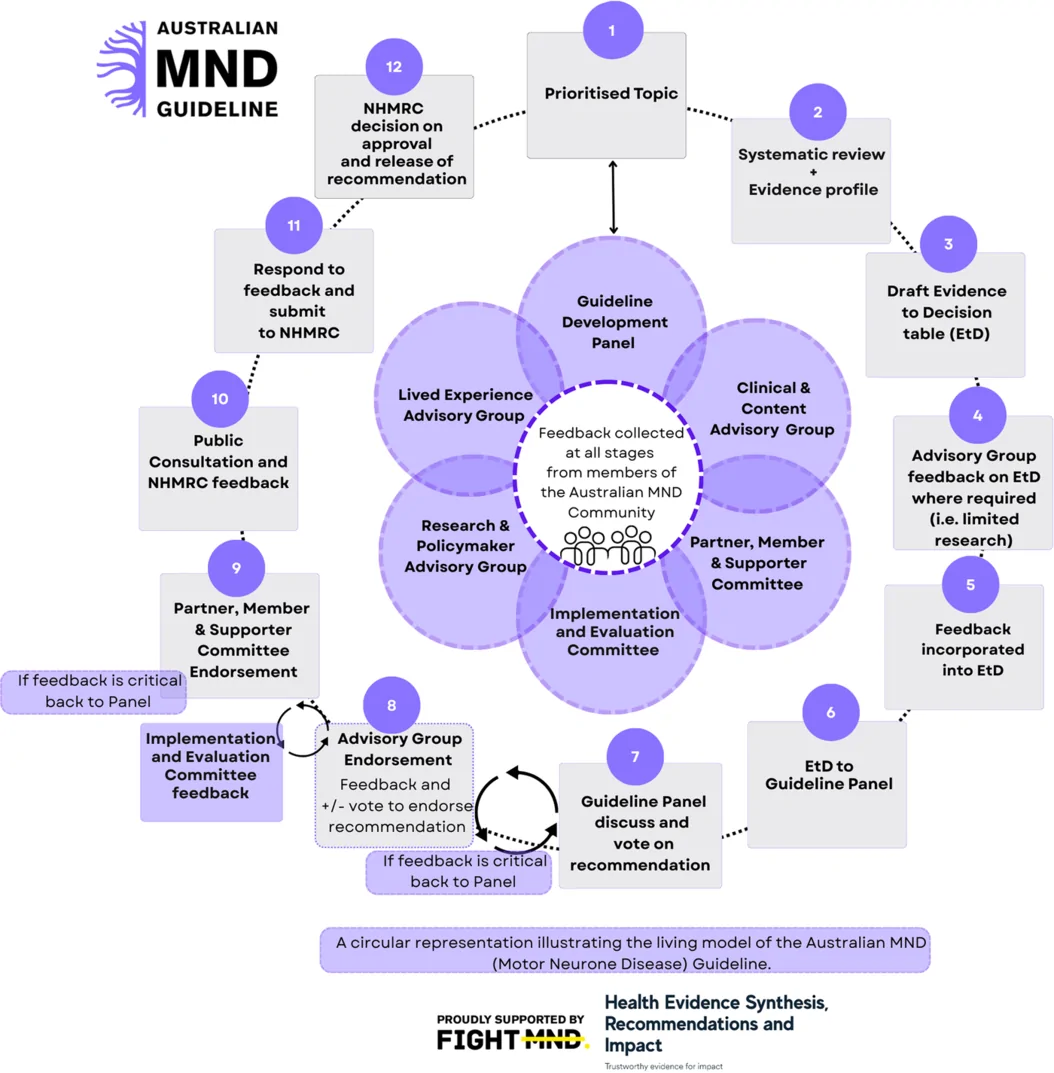
Australian Motor Neurone Disease (MND) Guideline development process.

For all questions included in the guideline, draft EtD Frameworks and recommendations will be prepared (either de novo or adapted/adopted) and the Guideline Panel will be asked to evaluate the evidence and make explicit judgements for each criteria. It is important to note that additional evidence can be generated to inform decision‐making. This may include not only systematic reviews but also primary research, such as qualitative studies, allowing EtD decisions to be supplemented by a diverse range of evidence beyond existing reviews. Based on their overall assessment across criteria, the Guideline Panel will reach a conclusion about the direction of their recommendation (for or against the intervention) and the strength of their recommendation [31]. Facilitated by the guideline methodologist, the panel will provide a justification for their recommendation, based on the criteria used in their assessment. Throughout this process, there will be multiple stages for input and feedback from all groups involved in the guideline. As outlined in Figure 3, the EtD framework for each guideline question will be circulated to all groups and committees in the governance structure, which collectively represent the Australian MND community (including people with lived experience of MND, carers, clinicians, allied health professionals, researchers, service providers, and policy makers). At each stage, feedback will be collected through structured online surveys and targeted meetings. Both quantitative survey responses and qualitative comments will be collated, analysed, and summarised for the Guideline Panel. The Panel will consider this synthesis and determine how feedback is incorporated, with critical feedback triggering additional feedback loops to ensure clarity and consensus before finalisation.

### 4.4 Implementation and Evaluation Planning

The Knowledge Translation, Dissemination, Implementation and Evaluation Committee will be established at the onset to ensure that translation and implementation of the guideline is considered from the beginning of the project and is not an afterthought. The Committee will include multidisciplinary representation, with members drawn from clinical practice, research, policy, and people with lived experience of MND to ensure that diverse perspectives inform decision‐making. In addition to reviewing the implementability of recommendations [23, 32], the Committee will codesign dissemination and translation strategies, including decision aids, multiple output formats, website content, promotional materials, and social media campaigns. Evaluation plans will be developed in parallel with guideline creation, including the identification of performance measures and quality standards, using rigorous and transparent methods [33, 34, 35]. Data on implementation and impact will be collected through interest holder feedback, monitoring of dissemination reach, and assessment of clinical practice change, with findings reported back to the Committee and governance groups to inform iterative improvement.

### 4.5 Transition to Living Guideline Model

The guideline will be designed to support future transition to a living guideline model. A living evidence map will be maintained to monitor new research and enable rapid updates to recommendations.

## 5 Conclusion

The development of an Australian MND guideline is both timely and critical. While international guidelines exist in MND care, local adaptation is necessary to reflect the specific needs, systems, and population diversity in Australia. This protocol outlines a robust, inclusive, and future‐focused approach to developing a guideline that is methodologically sound, contextually appropriate, and centred on the lived experience of people with MND.

## Author Contributions

**Cindy Stern:** writing – original draft, writing – review and editing. **Danielle Pollock:** conceptualisation, methodology, writing – original draft, writing – review and editing, funding acquisition. **Timothy Barker:** conceptualisation, methodology, writing – original draft, writing – review and editing, funding acquisition. **Grace McKenzie Holland:** conceptualisation, methodology, writing – original draft, funding acquisition. **Sabira Hasanoff:** conceptualisation, methodology, writing – original draft, writing – review and editing, funding acquisition. **Nipun Shrestha:** writing – review and editing. **Ines Semendric:** writing – review and editing. **Lemma Negesa Bulto:** writing – review and editing. **Sitasma Sharma:** writing – review and editing. **Anna Fragkoudi:** writing – review and editing. **Sebastian Cole Facchin‐Young:** writing – review and editing. **Abigail Molly Day‐Sharman:** writing – review and editing. **Lara Stollery:** writing – review and editing. **Jasmine Beasley‐Hall:** methodology, writing – review and editing, funding acquisition. **Camille Schubert:** methodology, writing – review and editing, funding acquisition. **Lynne Giles:** methodology, writing – review and editing, funding acquisition. **Tracy Merlin:** methodology, writing – review and editing, funding acquisition. **Steve Vucic:** writing – review and editing. **Jo Whitehouse:** writing – review and editing. **Zachary Munn:** conceptualisation, methodology, writing – original draft, writing – review and editing, funding acquisition.

## Ethics Statement

As this is a protocol for a guideline it does not require ethics approval.

## Conflicts of Interest

Cindy Stern, Grace McKenzie Holland, Ines Semendric, Lemma Bulto, Sitasma Sharma, Anna Fragkoudi, Nipun Shrestha and Danielle Pollock are members of the Adelaide GRADE Centre. Timothy Barker is Deputy Director of the Adelaide GRADE Centre. Zachary Munn is Director of the Adelaide GRADE Centre. Sabira Hasanoff is the Coordinator of the Adelaide GRADE Centre. This protocol is being conducted for the purposes of informing a guideline. The co‐chairs of the Guideline panel are authors of this review.

## Supporting information

Suppl.

## Data Availability

All data and files informing the guideline will be publicly available on a project space using open science platforms. The completed guideline will be available on MAGICapp.
