# Supplementary material for: Protocol for the Development of The Australian Motor Neurone Disease (MND) Guideline
Source: Clin Public Health Guidel. 2025 Dec 10;3(1):e70055. doi: 10.1002/gin2.70055 (PMC13563378; doi:10.1002/gin2.70055)
Supplement: Supplementary file 1 [file GIN2-3-e70055-s001.docx]

This manuscript contains no supplementary files. All is included in the manuscript.
